# Declining rates of surgery in pediatric-onset inflammatory bowel disease: a Swedish nationwide register-based cohort study 2002-2022

**DOI:** 10.1093/ecco-jcc/jjag127

**Published:** 2026-09-15

**Authors:** Helena Rolandsdotter, Kári Kristjánsson, Åsa H Everhov, Caroline Nordenvall, The Swibreg Study Group, Petter Malmborg, Pär Myrelid, Matilda Bräutigam, Jonas F Ludvigsson, Ola Olén

**Affiliations:** Department of Clinical Science and Education, Karolinska Institutet, Södersjukhuset, 118 83 Stockholm, Sweden; Division of Clinical Epidemiology, Department of Medicine Solna, Karolinska Institutet, 171 64 Stockholm, Sweden; Pediatric Gastroenterology and Nutrition Unit, Sachs’ Children and Youth Hospital, Södersjukhuset, 118 83 Stockholm, Sweden; Division of Clinical Epidemiology, Department of Medicine Solna, Karolinska Institutet, 171 64 Stockholm, Sweden; Department of Clinical Science and Education, Karolinska Institutet, Södersjukhuset, 118 83 Stockholm, Sweden; Division of Clinical Epidemiology, Department of Medicine Solna, Karolinska Institutet, 171 64 Stockholm, Sweden; Department of Molecular Medicine and Surgery, Karolinska Institutet, 171 76 Stockholm, Sweden; Department of Pelvic Cancer, Karolinska University Hospital, 171 76 Stockholm, Sweden; Department of Clinical Science and Education, Karolinska Institutet, Södersjukhuset, 118 83 Stockholm, Sweden; Division of Clinical Epidemiology, Department of Medicine Solna, Karolinska Institutet, 171 64 Stockholm, Sweden; Pediatric Gastroenterology and Nutrition Unit, Sachs’ Children and Youth Hospital, Södersjukhuset, 118 83 Stockholm, Sweden; Department of Biomedical and Clinical Sciences, Linköping University, 581 85 Linköping, Sweden; Clinical Department of Surgery in Linköping, Region Östergötland, 581 85 Linköping, Sweden; Department of Pediatric Surgery, Queen Silvia Children’s Hospital, Sahlgrenska University Hospital, 416 50 Gothenburg, Sweden; Institute of Clinical Sciences, Sahlgrenska Academy, Gothenburg University, 405 30 Gothenburg, Sweden; Department of Medical Epidemiology and Biostatistics, Karolinska Institutet, 171 65 Stockholm, Sweden; Department of Pediatrics, Orebro University Hospital, 703 82 Orebro, Sweden; Department of Clinical Science and Education, Karolinska Institutet, Södersjukhuset, 118 83 Stockholm, Sweden; Division of Clinical Epidemiology, Department of Medicine Solna, Karolinska Institutet, 171 64 Stockholm, Sweden; Pediatric Gastroenterology and Nutrition Unit, Sachs’ Children and Youth Hospital, Södersjukhuset, 118 83 Stockholm, Sweden

**Keywords:** intestinal surgery, children, advanced therapies

## Abstract

**Background and Aims:**

The use of advanced therapies in pediatric-onset inflammatory bowel disease (PIBD) has increased substantially over the past two decades, while corresponding changes in surgical risk remain insufficiently studied. We aimed to compare the use of PIBD-related surgery across calendar periods.

**Methods:**

Using nationwide, population-based registers in Sweden, we identified all patients with PIBD, including Crohn’s disease (CD) and ulcerative colitis (UC). We compared the cumulative incidence of IBD-related surgery by calendar period of first PIBD diagnosis (2002-2011 vs 2012-2022), with follow-up through adulthood. Trends in the use of advanced therapies were described for the same periods.

**Results:**

We identified 8200 patients with PIBD, with a median follow-up of 13.9 years for those diagnosed in 2002-2011 (*n* = 3751) and 4.3 years for 2012-2022 (*n* = 4449). The 5-year cumulative incidence of luminal surgical intervention decreased over time (9.6% vs 6.1%, *P* < .001). This reduction was observed for colectomy in UC (*P* = .044) but not in CD (*P* = .121). Patients with CD showed marked declines in small bowel surgery, including ileocecal resections, and in segmental colonic resections (*P* < .001 and *P* = .006). Between the first and second calendar period, the annual proportion of patients starting advanced therapies increased 2-fold for first-line use and 4-fold for second-line use, while third-line therapy was only observed in the later period.

**Conclusion:**

Over the past two decades, the cumulative incidence of several types of IBD-related surgery in PIBD has declined in parallel with a pronounced increase in the use of advanced therapies.

## Graphical abstract

**Graphical Abstract:**
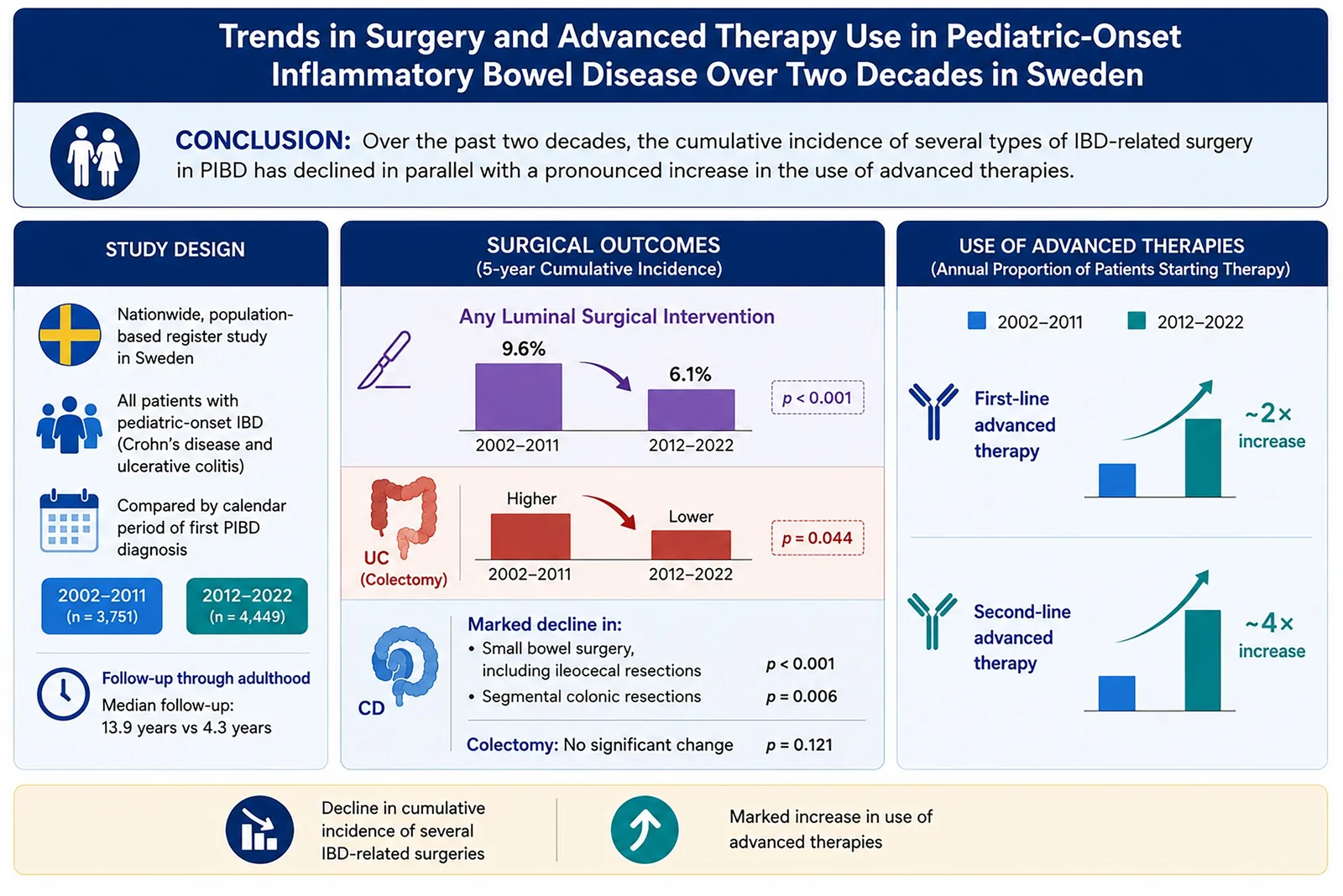

## 1. Introduction

Inflammatory bowel disease (IBD) affects all ages and has now reached an overall population prevalence of around 1% in many high-income countries.^1^^,^^2^ The global incidence of pediatric-onset IBD (PIBD) is rising^3^^,^^4^ and it is estimated that approximately 10% of all patients with IBD in northern Europe and Scandinavia are diagnosed during childhood. Compared to the adult-onset IBD phenotype, children with IBD more often experience severe and extensive disease.^5^^,^^6^ Conventional medical therapies for PIBD include aminosalicylate preparations, corticosteroids, and immunomodulators along with various dietary interventions in Crohn’s disease (CD).^7–9^ Over recent decades, advanced therapies, ie, biologic treatments and small molecules therapies, have offered promising potential for improving therapeutic outcomes in PIBD (Appendix, Figure S1). Nevertheless, IBD-associated surgery is still ­sometimes needed. In pediatric ulcerative colitis (UC) and colonic CD, colectomy may be required for uncontrolled disease, disease complications, or rarely because of colorectal cancer,^10^^,^^11^ while children with CD may also need different types of bowel resections, diverting stoma or perianal surgery in addition to their medical therapy.^7^^,^^12^

Several studies with varying designs have investigated the use of surgery over time in routine IBD care, yielding conflicting results. Some studies (mainly including adult patients) have indicated that CD-associated surgery has declined over the years, which in turn has been assumed to be an effect of increased awareness of the risks associated with extensive surgery in CD patients (ie, multiple resections may be associated with a risk for short-bowel syndrome) and the introduction of newer, advanced therapies.^13–17^ Pediatric IBD studies from Denmark (with follow-up until 2013), Sweden (2014), and Israel (2019) have hitherto found no association between use of IBD-surgery and calendar period.^18–20^ However, with steadily increasing use of advanced therapies, this is a rapidly moving target and modern data on PIBD surgery rates over the past decade remain scarce.

We therefore conducted a population-based nationwide cohort study with follow-up data until December 31, 2022, to assess how the cumulative incidence of IBD-related surgery in PIBD has changed over the last two decades.

## 2. Methods

### 2.1. Study design

In this nationwide, population-based cohort study, using mandatory registers with prospectively collected data from routine medical practice, we compared the cumulative incidence of various types of IBD-related surgeries performed in PIBD with a first ever diagnosis of IBD in the calendar periods 2002-2011 and 2012-2022.

### 2.2. Ethical permission

The study was approved by the Regional Ethics Committee in Stockholm (Dnr-2021-06209-01, 2022-04384-02 and 2023-04868-02). Individual informed consent was not required in this study, based on pseudonymized register data.

### 2.3. Setting and data sources

In Sweden, the healthcare system is almost exclusively funded through taxation, ensuring that all citizens have equal access to both acute and elective care.^21^ Swedish citizens are assigned a unique personal identity number, which is used in all interactions with the healthcare system and enables linkage of registers.^22^ The collected data include information on diagnoses, morbidity, mortality, as well as emigration, with minimal loss to follow-up.^23^ We used the Swedish National Patient Register (NPR),^24^^,^^25^ the Prescribed Drug Register,^26^^,^^27^ the Total Population Register,^28^ and Swedish Inflammatory Bowel Disease Register (SWIBREG)^29^ to find the baseline and follow-up data including patient demographics, IBD subtype, IBD phenotype (eg, extent in UC and location in CD), treatments and outcomes (Appendix, Tables S1 and S2a).

### 2.4. Study population

We used validated definitions to identify Swedish children in the NPR, diagnosed with CD, UC or IBD-U^30–32^ before their 18^th^ birthday between 1 Jan 2002—31 Dec 2022. The included patients were required to have at least two diagnostic listings of IBD at two distinct time points, as identified by the International Classification of Diseases tenth revision (ICD-10) codes in the Swedish NPR. If the first two diagnostic codes for IBD subtypes were identical (either CD, UC, or inflammatory bowel disease unclassified [IBDU-), the patient was classified accordingly. If the first two codes represented different subtypes, the patient was classified as having IBDU (Appendix, Table S2a).

### 2.5. Surgery

We assessed the incidence of IBD-associated surgery from date of first listing of IBD. The IBD-related surgeries included in this study were colectomy, small bowel resection (including ileocecal resection), segmental colonic resection, perianal surgery, other intestine surgery, and reconstructive surgery inclusive stoma-surgery. We employed validated definitions, including the distinction between surgery types, based on the Nordic Medico-Statistics Committee (NOMESCO) Classification of Surgical Procedures, as recorded in the NPR (Appendix, Table S3^33^). Some children underwent colectomy, other bowel resection, or perianal surgery before the first IBD diagnosis. The majority were operated on close to the IBD diagnosis (data not shown). The date of any IBD-associated surgery performed within 6 months prior to the first diagnostic listing of IBD was regarded as the first diagnostic listing of IBD and included as an IBD outcome.

### 2.6. Exposures to IBD drugs

We used the Swedish Prescribed Drug Register to capture data on all dispensed prescription medications (eg, corticosteroids, aminosalicylates, immunomodulators, advanced therapies [small molecules and biological treatments]). For advanced therapies administered as infusions in hospital settings we also used data from NPR and SWIBREG.^27^ All relevant Anatomical Therapeutic Chemical classification codes are listed in Appendix, Table S4.

### 2.7. Statistics

The cumulative incidence was calculated for (1) luminal surgery, (2) colectomy, (3) segmental small bowel resection including ileocecal resection, (4) segmental colonic resection, (5) perianal surgery, (6) any IBD-surgery, and (7) stoma-surgery. Given the aim to describe all surgeries during the course of PIBD and given the many surgeries very early in the disease course, follow-up time was set from the first diagnosis of IBD (or surgery just before the first IBD diagnostic listing), rather than at the somewhat later date when the patient fulfilled our PIBD definition. The resulting immortal time was not deemed to create any meaningful bias across the compared time periods. The patients were followed until the date of the specific surgery of interest (irrespective of whether other IBD-associated surgery had been performed), death, emigration, or December 31, 2022, whichever occurred first. The cumulative incidence of surgery was estimated using the Kaplan–Meier method, which appropriately accounts for censoring, so the 5-year estimate remains statistically valid even though only a minority of the later cohort reached 5 years of follow-up. Differences in time to surgery between diagnosis periods (2002-2011 and 2012-2022) were assessed using the log-rank test. We also compared the cumulative incidence of the two groups at 1, 3, and 5 years.

We also performed several sensitivity analyses: (1) we produced results based on IBD subtype (CD vs UC) based on all information at the end of follow-up (instead of only the two first diagnostic listings); (2) we truncated analyses at December 31, 2019 to ensure that any potential decline in luminal IBD-surgery was not due to the Covid-19 pandemic; and (3) we calculated cumulative incidence of colectomy restricted to patients with colitis (extent 3–4 in UC and location 2 in CD, according to the Paris classification).

Cox proportional hazards regression was used to analyze time to surgery, with time since first IBD diagnosis as the underlying time scale. Follow-up started at the date of first IBD diagnosis and ended at the date of surgery, death, emigration, or end of follow-up, whichever occurred first. Due to violations of the proportional hazard assumption in some analyses, stratified Cox regression models were applied. Stratification variables differed by analysis and included diagnosis period, sex, age at diagnosis, and IBD subtype, depending on the outcome of interest. Covariates not used for stratification were included as model predictors. The proportional hazard assumptions were assessed using Schoenfeld residuals. All statistical analyses were performed using R v.4.4.1 (R Core Team, 2024). Statistical tests were two-sided, with significance level of α < .05.

## 3. Results

### 3.1. Cohort characteristics at IBD diagnosis

We identified 8200 children diagnosed with PIBD. The children were largely similar in terms of sex and age distribution at diagnosis across the two calendar periods (2002-2011 and 2012-2022, Table 1).

**Table 1.** Patient characteristic at the time of first diagnosis in 8200 patients with incident pediatric-onset inflammatory bowel disease (PIBD) by calendar period.

		2002-2011	2012-2022
**Total patients (*n*, %)**	3751 (100%)		4449 (100%)
**Males (*n*, %)**	2084 (55.5%)		2513 (56.5%)
**Females (*n*, %)**	1667 (44.5%)		1936 (43.5%)
**Age, years**			
**Mean age (SD)**	14 (4)		14 (4)
**Median age (range)**	15 (0-18)		15 (0-18)
**<6 years (*n*, %)**	186 (5%)		203 (4,5%)
**6-9 years (*n*, %)**	334 (9%)		451 (10%)
**10-14 years (*n*, %)**	1490 (40%)		1728 (39%)
**15-17 years (*n*, %)**	1741 (46%)		2067 (46%)
**IBD subtype**			
**UC (*n*, %)**	1739 (46%)		1735 (39%)
**CD (*n*, %)**	1607 (43%)		1897 (43%)
**IBDU (*n*, %)**	405 (11%)		817 (18%)
**Country of birth**			
**Sweden**	3576 (95%)		4036 (91%)
**Nordic countries (except Sweden)**	18 (0.5%)		31 (0.7%)
**Non-Nordic countries**	157 (4%)		381 (9%)
**Comorbidity at start of follow-up**			
**Ankylosing spondylitis**	1 (0.03%)		4 (0.1%)
**Cerebrovascular disease**	2 (0.1%)		7 (0.2%)
**Chronic obstructive pulmonary disease**	3 (0.1%)		9 (0.2%)
**Diabetes mellitus**	17 (0.5%)		30 (0.7%)
**Psoriasis arthritis**	4 (0.1%)		5 (0.1%)
**Rheumatoid arthritis**	4 (0.1%)		7 (0.2%)
**Systemic lupus erythematosus**	5 (0.1%)		3 (0.1%)
**Primary sclerosing cholangitis**	42 (1%)		32 (1%)
**Other extraintestinal manifestations**	149 (4%)		230 (5%)

Abbreviations: SD, standard deviation; UC, ulcerative colitis; CD, Crohn’s disease; IBDU, inflammatory bowel disease unclassified.

### 3.2. Cohort characteristics at end of follow-up

At the end of follow-up, the median follow-up duration was 13.9 years (range 0–21 years) for patients diagnosed 2002-2011, and 4.3 years (range 0–11 years) for patients diagnosed 2012-2022. Exposure to advanced therapies within 5 years of follow-up more than doubled during the second calendar period (Appendix, Table S5). Most surgical interventions were performed during adolescence, with a median age at first surgery of 17 years (range 0–35) (Appendix, Figure S2).

### 3.3. Surgery

**Figure 1.**
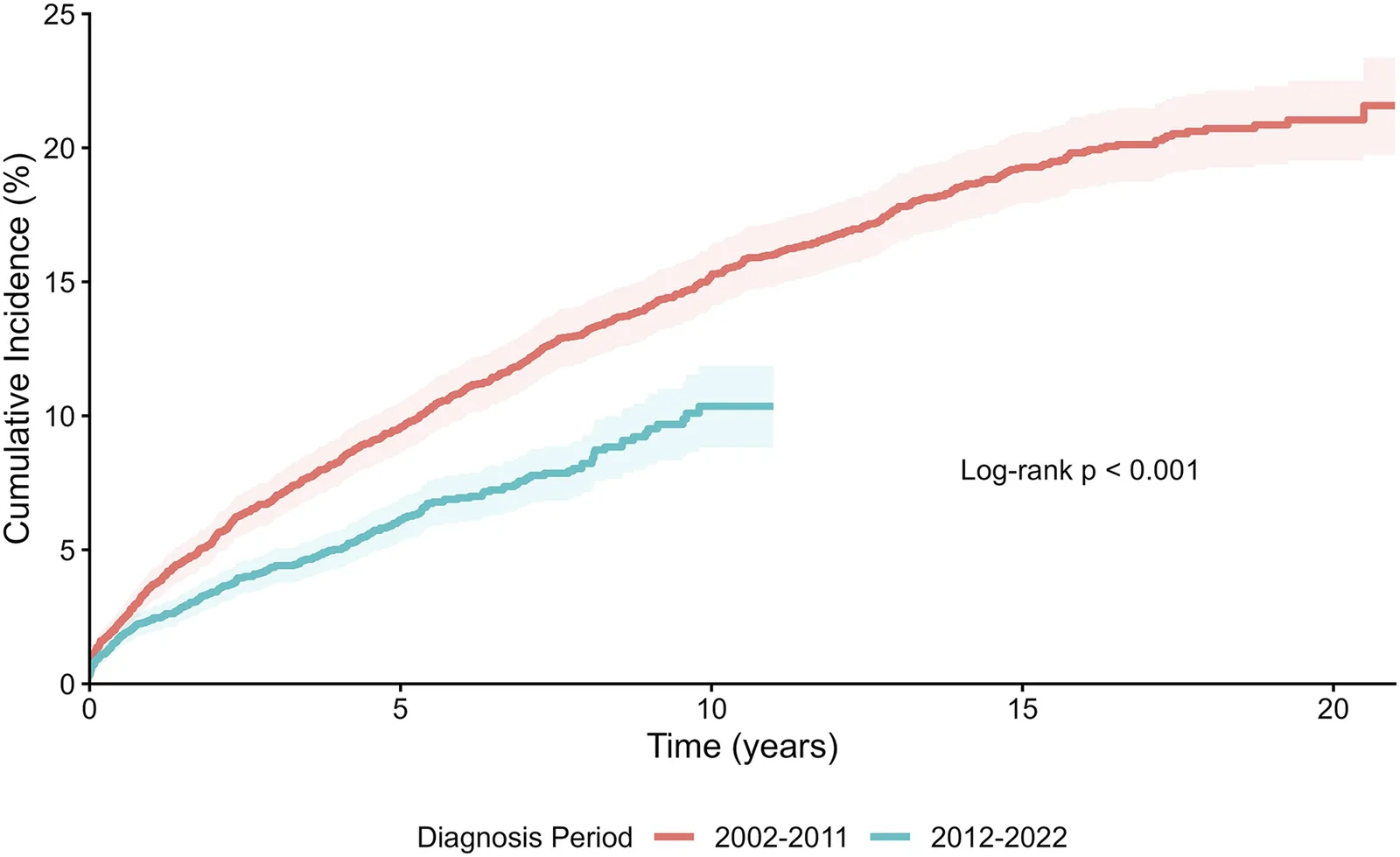
The cumulative incidence, with 95% confidence intervals, of luminal surgery, in pediatric-onset inflammatory bowel disease (IBD) by calendar period of first IBD diagnosis.

**Figure 2.**
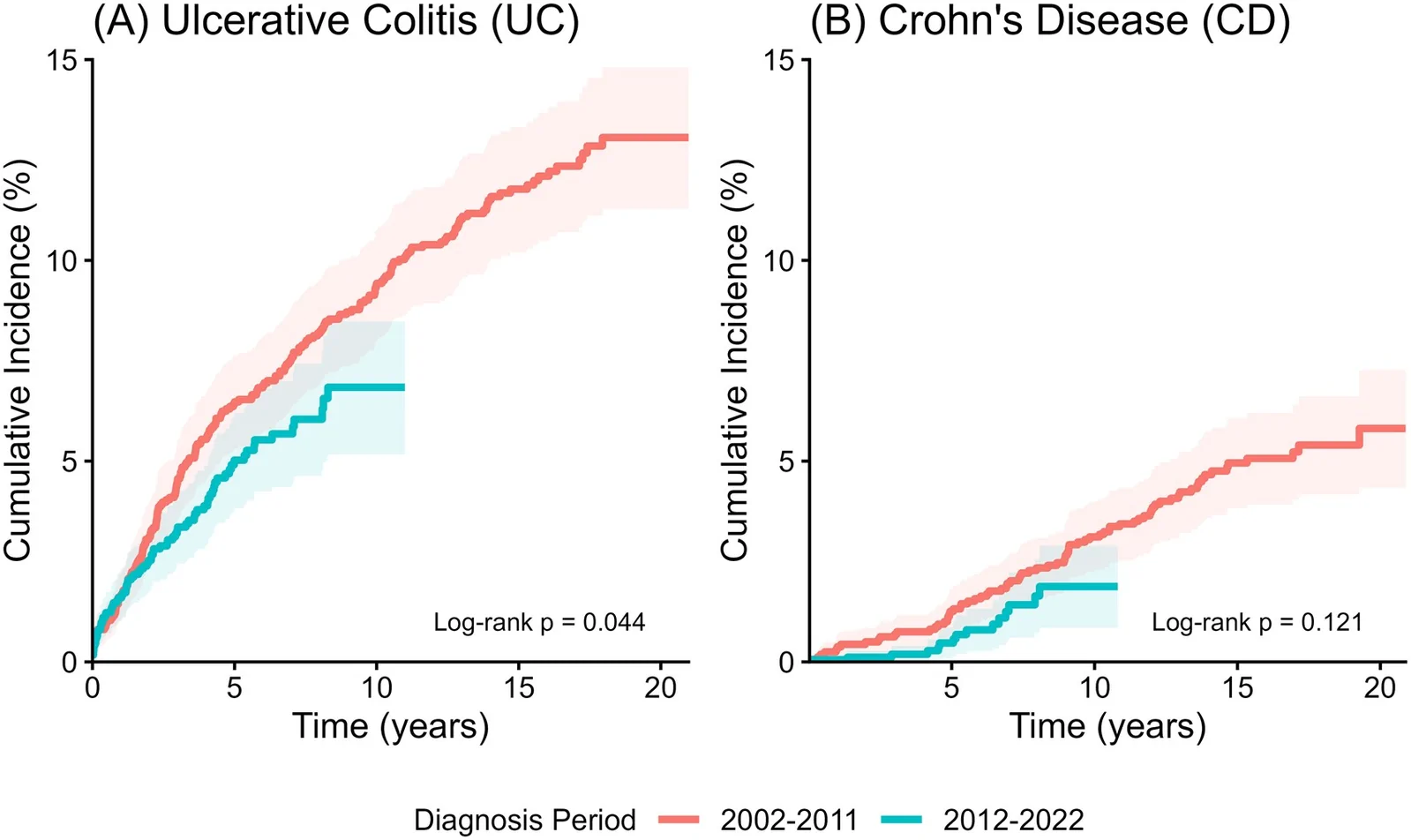
(A, B) The cumulative incidence, with 95% confidence intervals, of colectomy in pediatric-onset ulcerative colitis and Crohn’s disease by calendar periods.

In total, 18% of the 8200 patients during the whole time-period (2002-2022) underwent some kind of IBD-associated surgery (Appendix, Table S6). The cumulative incidence curves of luminal surgery separated after approximately a year of follow-up and the 5-year cumulative incidence decreased between calendar periods from 9.6% in 2002-2011 to 6.1% in 2012-2022 (*P* < .001, Figure 1, Table 2). We also investigated the 5-year cumulative incidence of colectomies and found a significant decrease in the later period in UC (6.4% in the earlier compared with 5% in the latter, *P* = .044) but not in colectomies in CD, although absence of statistical significance most likely reflects the small number of colectomies in CD (1.3% in the earlier and 0.5% in the later cohort, *P* = .121, Figure 2A, B, Table 2).

**Table 2.** Cumulative incidence of different types of surgery at 1, 3, and 5 years in pediatric-onset Crohn’s disease and ulcerative colitis (with follow-up beyond 18th birthday) by calendar period of first inflammatory bowel disease diagnosis.

	Period	1 year	3 years	5 years	*P*-value^a^
**Crohn’s disease**					
**Colectomy**	2002-2011	0.4%	0.6%	1.3%	.121
	2012-2022	0.1%	0.2%	0.5%	
**Small bowel resection incl. ileocecal resection**	2002-2011	4.8%	7.2%	9.7%	<.001
	2012-2022	2.6%	4.2%	5.6%	
**Segmental colonic resection**	2002-2011	0.9%	1.2%	1.4%	.006
	2012-2022	0.3%	0.4%	0.8%	
**Perianal surgery**	2002-2011	6.0%	8.5%	10.4%	.642
	2012-2022	7.4%	8.9%	10.1%	
**Ulcerative colitis**					
**Colectomy**	2002-2011	1.6%	4.6%	6.4 %	.044
	2012-2022	1.6%	3.4%	5.0%	
**Crohn’s disease *and* ulcerative colitis**					
**Any surgery**	2002-2011 2012-2022	6.3% 5.8%	10.7% 8.5%	14.0% 10.9%	<.001
**Luminal surgery**	2002-2011	3.7%	7.0%	9.6 %	<.001
	2012-2022	2.4%	4.4%	6.1%	
**Formation of ileostoma**	2002-2011 2012-2022	1.2% 1.2%	3.1% 2.3%	4.4% 3.4%	.008
**Formation of colostoma**	2002-2011 2012-2022	0.1% 0.1%	0.2% 0.4%	0.5% 0.5%	.833
**Closure of stoma** ^b^	2002-2011 2012-2022	29.2% 28.2%	49.7% 57.2%	59.0% 62.3%	.396

a *P*-values based on log-rank tests.

b Proportion of all patients undergoing formation of stoma.

**Figure 3.**
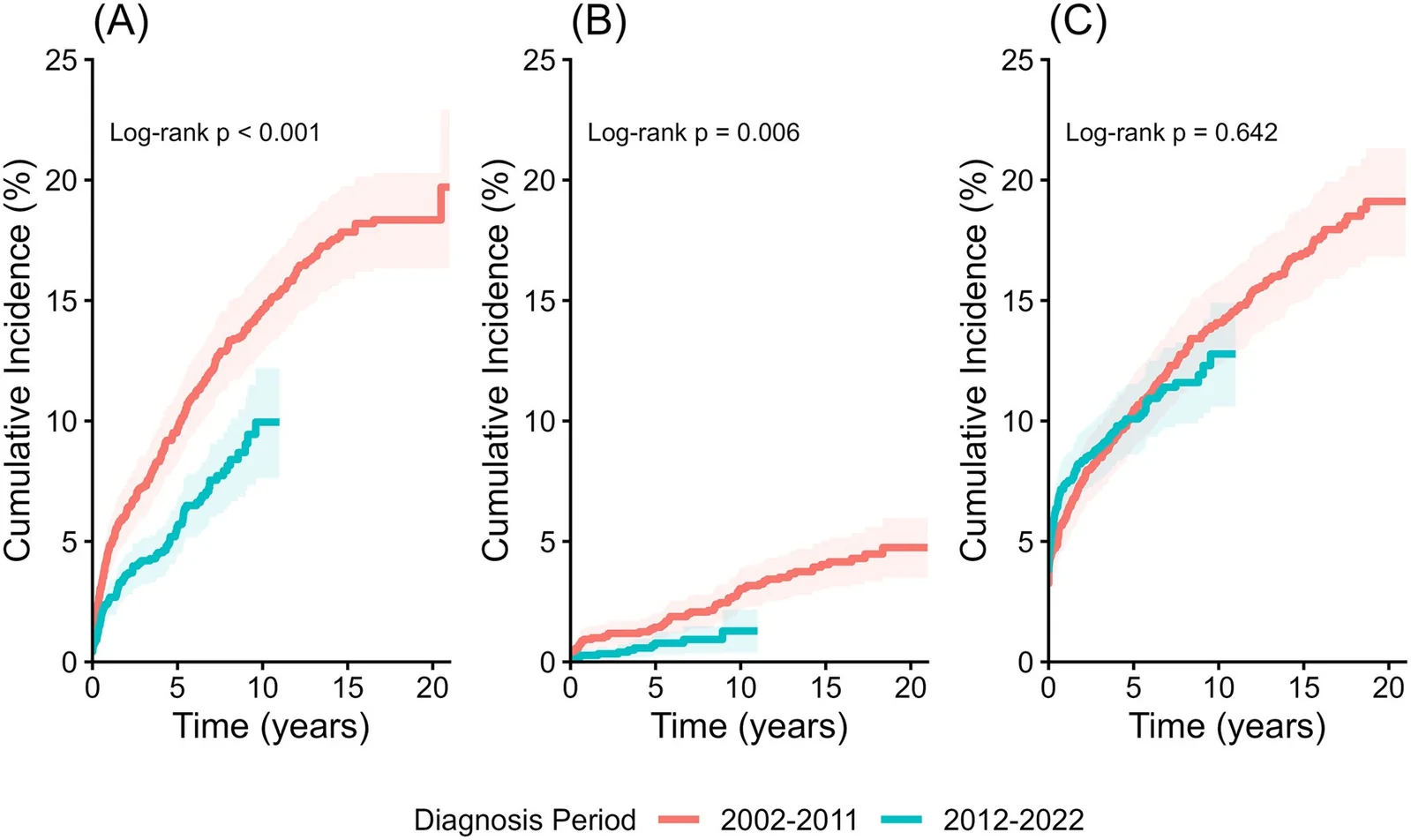
(A–C) The cumulative incidence, with 95% confidence intervals, of (A) small bowel resection (including ileocecal resections), (B) segmental colonic resection, and (C) perianal surgery in children with Crohn’s disease by calendar period of first Crohn’s disease diagnosis.

**Figure 4.**
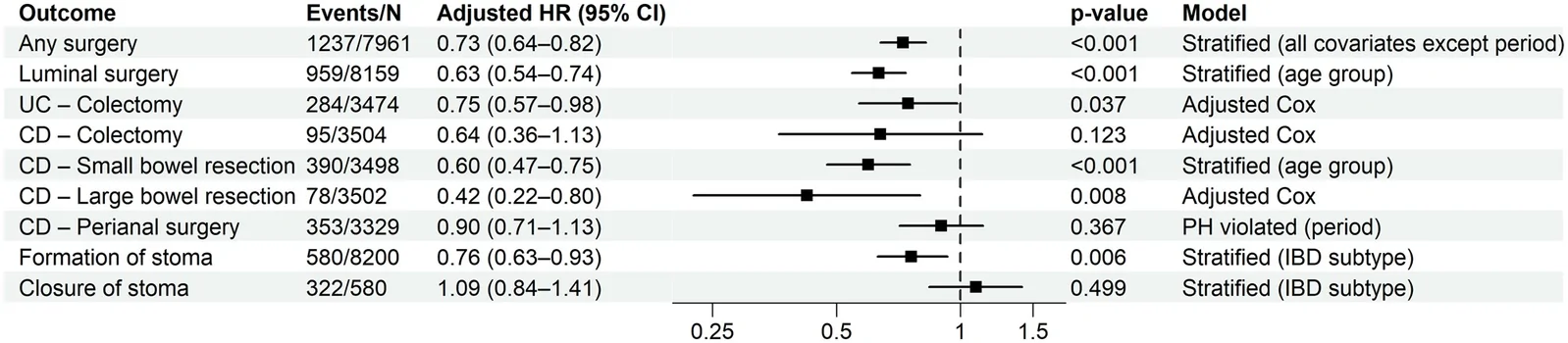
Adjusted hazard ratios for surgical outcomes after pediatric-onset inflammatory bowel disease, estimated using Cox proportional hazards models adjusted for sex and age at diagnosis. Model specification varied by outcome, including stratification where appropriate.

The 5-year cumulative incidence of small bowel resection (including ileocecal resection) and segmental colonic resections in CD decreased significantly in the later calendar period (Figure 3A, B, Table 2), while the cumulative incidence of CD perianal surgery was not statistically different across calendar periods (Figure 3C, Table 2). Additionally, when using Cox-regression analysis to compare the two time-periods we found the same pattern with significant decreases in colectomies in UC (hazard ratio [HR] 0.75, 95% confidence interval [CI]: 0.57-0.98), as well as in small bowel resections (HR 0.60, 95% CI: 0.47-0.75) and in large bowel resections in CD (HR 0.42, 95% CI: 0.22-0.80, Figure 4).

**Figure 5.**
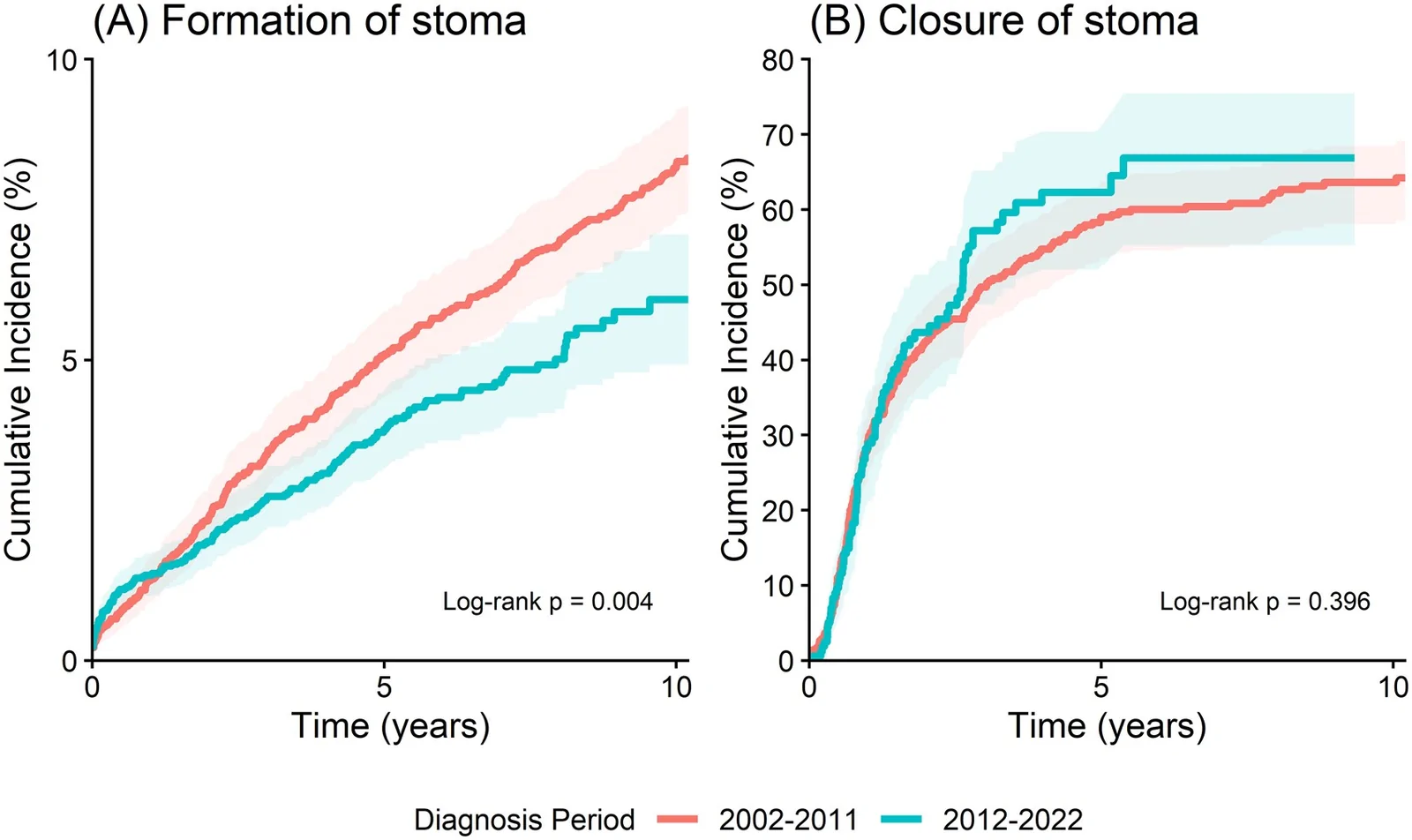
Cumulative incidence curves, with 95% confidence intervals, of formation of stoma (A) and closure of stoma (B) in children with inflammatory bowel disease (2002-2011 and 2012-2022).

We also investigated reconstructive surgical procedures and found that ileorectal anastomosis was considerably more common than ileal pouch–anal anastomosis (Appendix, Table S6). Furthermore, a statistically significant reduction in the cumulative incidence of stoma formation was seen, declining from 5.1% in the earlier time period to 3.9% in the later period (Figure 5). However, that difference was observed in the rate of ileostomy formation, while no significant differences were identified between the two time periods with respect to the incidence of colostomy formation, nor in the stoma closure procedures (Table 2, Appendix, Figure S3A–C).

Boys with CD had a significantly higher risk of perianal surgery compared to girls (HR 1.45, 95% CI: 1.17-1.81), whereas individuals diagnosed before the age of 6 years had a significantly lower relative risk than patients diagnosed as teenagers (HR 0.38, 95% CI: 0.15-0.92) (Appendix, Figure S4).

### 3.4. Advanced therapies

**Figure 6.**
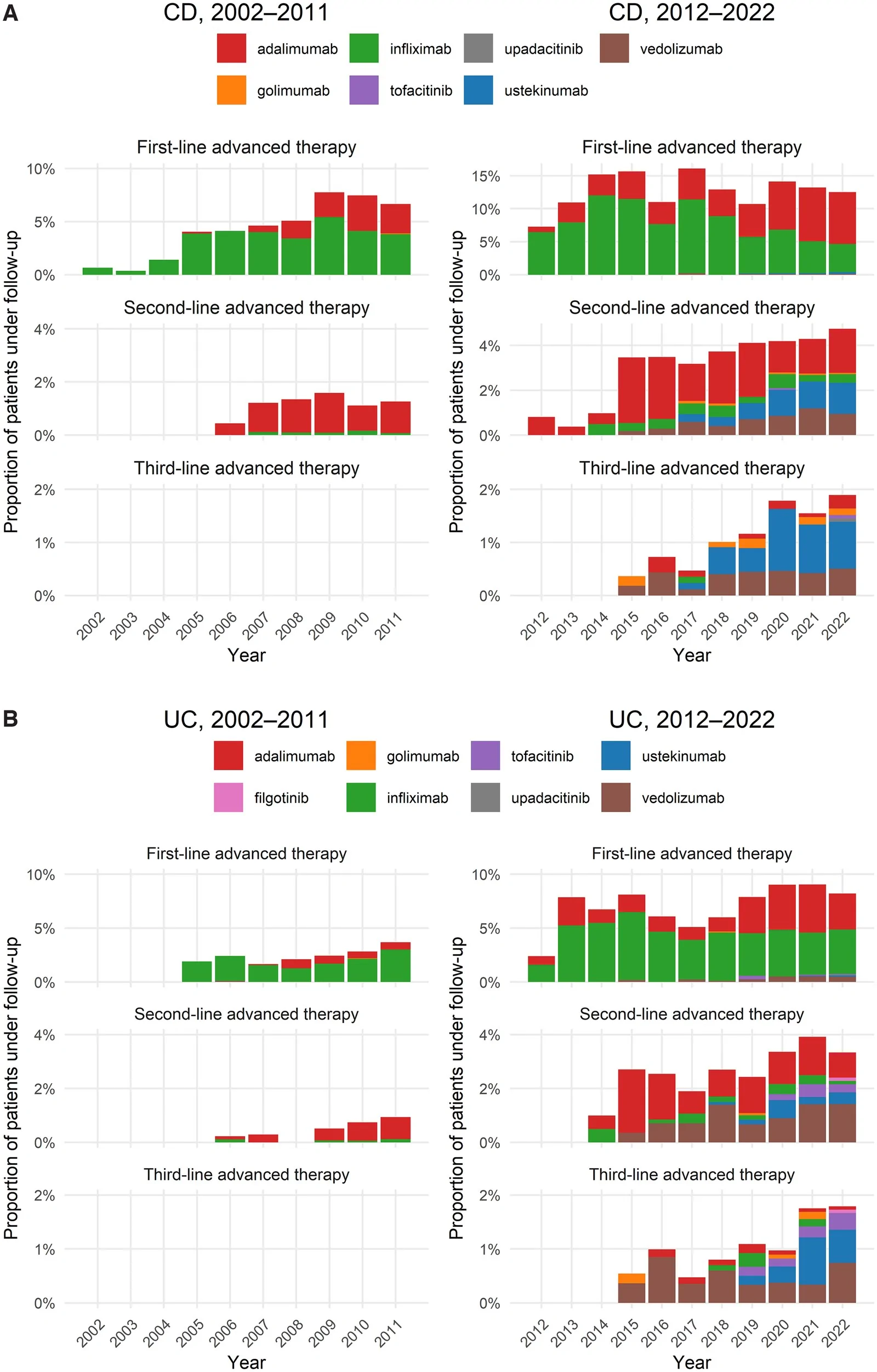
(A) Annual proportion of patients with pediatric-onset Crohn’s disease (CD) starting an advanced therapy (first, second, and third line) across calendar periods (2002-2011 vs 2012-2022*). *SWIBREG and the Prescribed Drug Register started in 2005, and therefore data on drugs before 2005 were retrieved from NPR, which had suboptimal coverage for infusion drugs at the time. (B) Annual proportion of patients with pediatric-onset ulcerative colitis (UC) starting an advanced therapy (first, second, and third line) across calendar periods (2002-2011 vs 2012-2022*). *SWIBREG and the Prescribed Drug Register started in 2005, and therefore data on drugs before 2005 were retrieved from NPR, which had suboptimal coverage for infusion drugs at the time.

The annual number of PIBD patients initiating different lines of advanced therapy increased dramatically from 2002-2011 to 2012-2022 (Figure 6A, B). During the first calendar period, only infliximab and adalimumab were available (Appendix, Figure S1). During the second calendar period, the annual number of patients initiating infliximab and adalimumab was much higher and an increasing number of patients were treated with second-generation biologics and small molecule therapies (eg, vedolizumab, ustekinumab, tofacitinib, Figure 6A, B). This finding is supported by the nearly 4-fold increase in the proportion of individuals receiving at least two advanced therapies prior to surgery in the later cohort compared with the earlier cohort, when analyses were restricted to individuals with at least five years of potential follow-up in both cohorts (6.3% in the 2002-2011 cohort and 21% in the 2012-2022 cohort) (Appendix, Table S7).

### 3.5. Sensitivity analyses

When we created versions of Figures 2 and 3 in which subtype information (UC and CD status) was based on all information at the end of follow-up, we found no meaningful changes to the results (Appendix, Figures S5 and S6). When we stopped follow-up on December 31, 2019 (to assess to what extent the Covid-19 pandemic might have influenced our findings), the difference between calendar periods for luminal surgery (as in Figure 1) remained unchanged (*P* < .001, Appendix, Figure S7). In versions of Table 2 and Figure 2 (colectomies) restricted to patients with total UC or Crohn’s colitis (E3-4 or L2 according to the Paris classification) at the start of follow-up, the number of individuals possible to include diminished, the cumulative incidence at 1, 3, and 5 years increased for both UC and CD compared to Figure 2, and although time-trends seemed similar, they were no longer statistically significant (Appendix, Figure S8, Table S8).

## 4. Discussion

### 4.1. Main findings

This Swedish national cohort study, which included 8200 patients with incident PIBD with follow-up through adulthood, demonstrated clearly reduced use of several types of IBD-associated surgery when comparing incident PIBD patients from 2002-2011 and 2012-2022. The 5-year cumulative incidence decreased significantly for luminal surgery overall, for colectomy in UC, and for segmental colonic and small bowel resections in CD, while the cumulative incidence of colectomy and of perianal surgery in CD did not change significantly.

### 4.2. Comparisons with previous findings

Previous studies on surgery rates in IBD have reported conflicting results, with few papers focussing on pediatric-onset IBD. Atia et al. reported stable rates of surgery in UC patients of all ages during the biologic era (2005-2019)^20^ and almost a decade ago, Nordenvall et al. also showed that calendar period of PIBD diagnosis was not associated with cumulative incidence of surgery (2002-2014).^19^ In contrast, Kalman et al. demonstrated that the 5-year cumulative incidence of first bowel surgery for CD of any age had significantly decreased (albeit very little) between 2002-2008 and 2009-2014.^17^ Conversely, Larsen et al. reported that, while surgery rates for UC decreased, no change in CD surgery could be seen when comparing two PIBD cohorts; 1998-2008 and 2009-2013.^18^

### 4.3. Strengths and limitations

To the best of our knowledge, this is the largest and most recent study to date on surgery in PIBD. A major strength is that the study took advantage of compulsory nationwide registers, containing data reported from routine medical practice in all specialized in- and outpatient care in Sweden, ensuring complete patient coverage. The definitions used to identify PIBD cases and the IBD-associated surgeries are reliable and validated.^30^^,^^33^ We also believe the results can be generalized to other high-income countries with similar capacity to provide advanced IBD treatments.

A limitation of the study is the absence of detailed clinical information, including data on disease activity, the extent of inflammation, the effects of various drugs, and the specific reasons for choosing or abstaining from surgery. Additionally, there is a slight risk of mis-classification of IBD subtypes as it has previously been shown that IBD subtypes change during follow-up and that misclassification of the disease extent or location may be seen in both UC and CD.^31^^,^^32^ However, we do not believe that this explains our main findings, since sensitivity analyses performed with subtype defined by the last diagnostic code before surgery or end of follow-up were almost identical to what is presented in this paper.

### 4.4. Potential mechanisms/explanations

Our finding that several types of surgical interventions for PIBD have decreased significantly could to some extent be due to a trend of decreasing confidence in surgical solutions,^13^ unrelated to pharmacological advancements. However, we think that the simultaneous dramatically increased use of advanced therapies (likely followed by better disease control) over the last decades may be a much more plausible and/or important explanation. In addition to increasing use of advanced therapies, the increasing adoption of therapeutic drug monitoring (TDM) with drug optimization is likely to have improved treatment success rates even more by facilitating personalized drug adjustments,^7^^,^^34^^,^^35^ which may further have contributed to a declining use of surgery in IBD over the past decade.

Our study demonstrated no reduced cumulative incidence of perianal surgery. This was, however, somewhat expected, since many of the perianal interventions included in our definition of perianal surgery are also part of the diagnostic or first therapeutic efforts for patients with such phenotypes. We unfortunately did not have enough information to be able to say whether perianal treatment success has increased in parallel with increased use of advanced therapies, although that is likely. Consistent with previous findings, our study indicated a significantly higher risk of perianal surgery in boys compared to girls.^36^^,^^37^ However, the reasons underlying the association between male sex and increased rates of perianal surgery remain poorly understood.^37^^,^^38^ Contrary to our earlier Swedish results with smaller sample size and much shorter follow-up time, we did not observe a higher colectomy rate in UC in boys.^19^ Further, we found that ileorectal anastomosis was a much more common reconstructive approach than ileal pouch anal anastomosis, albeit based on small numbers, which is in line with traditions of reconstructive surgery for this age group in Sweden.^39^

### 4.5. Clinical implications

Prognosis is a critical concern for patients diagnosed with PIBD and common questions from both patients and their families include what outcomes may arise if medical treatment proves ineffective,^29^ whether surgical intervention will be necessary, and the risk of complications from surgery. Updated real-world estimates of cumulative incidence for different types of surgical procedures are therefore crucial for these kinds of conversations with patients and caregivers.

An informed discussion about the prognosis of PIBD must encompass a balanced evaluation of the advantages and disadvantages of surgical intervention compared to advanced therapies (which are frequently used off-label in pediatric patients^40^). The retrospective long-term follow-up of the LIR! C study found early ileocecal resection in adult CD patients with isolated ileocecal disease to be a possible alternative to treatment with early infliximab,^41^ although whether this is true also in routine medical practice is still an open question.^42^ In the LIR! C trial, early ileocecal resection was safe and cost-effective compared to anti-TNF (before the advent of biosimilars, ie, when anti-TNF was still very expensive), suggesting that the same might be true when comparing surgery with other new and often very expensive advanced therapies.^43^^,^^44^ A related question is whether the timing and intensity of advanced therapy influence the subsequent need for surgery. Although an increasing proportion of patients were exposed to one or more advanced therapies before surgery over the study period (Appendix, Table S7), these data cannot establish whether earlier or more intensive treatment reduces or merely defers ­surgery, since such comparisons are confounded by indication—patients treated earlier and more intensively typically having more severe disease—and are susceptible to immortal-time bias. Disentangling this will require randomized trials such as LIR! C, or observational studies specifically designed to emulate them using detailed phenotypic and disease-activity data.

While this study clearly shows that many types of PIBD-associated surgery were performed later, we cannot (yet) know whether the life-time risk of surgery has changed in the studied cohorts. Another important question in this context is whether delayed surgery, with prolonged exposure to off-label (in the pediatric setting) immunosuppressive drugs with uncertain long-term effects might result in increased other risks, such as neoplasia development^45^—at least in patients who do not achieve simultaneous deep remission.^46^

## 5. Conclusion

For the last two decades, the cumulative incidence of several types of surgery has decreased in PIBD (colonic and small bowel resections in CD and colectomies in UC) while rates of colectomies and perianal surgery in CD have not. During the same period use of advanced therapies in PIBD has increased dramatically. Whether the risk of IBD-related surgery has truly decreased or has only been postponed remains to be seen in future long-time follow-up studies.

## Supplementary Material

jjag127_Supplementary_Data

## Data Availability

The data underlying this article cannot be shared publicly due to Swedish legislation.

## The SWIBREG-study group

Hans Strid^a^, Susanna Jäghult^b^, Jonas Halfvarson^c^, Charlotte Hedin^d^, Olof Grip^e^, Ulrika L. Fagerberg^f^, Karl Mårild^g^, Jóhann P. Hreinsson^j^, Henrik Hjortswang^k^, and Martin Rejler^l,m^

^n^Pär Myrelid, ^o^Caroline Nordenvall and ^p^Ola Olén

^a,d^Department of medicine Solna, Karolinska Institutet, Stockholm, Sweden and Karolinska University Hospital, Stockholm, Sweden

^b^Department of Clinical Science and Education, Södersjukhuset, Stockholm, Sweden

^c^Faculty of Medicine, Örebro University and Department of Gastroenterology, Örebro University Hospital, Sweden

^e^Department of Gastroenterology, Skåne University Hospital, Malmö, Sweden

^f^Department of Women’s and Children’s Health, Karolinska Institutet, Stockholm and Center for Innovation, Research and Education, Region Västmanland, Västerås, Sweden

^g^Department of Pediatrics, Institute of Clinical Sciences, Sahlgrenska Academy, Gothenburg, Sweden and Department of Gastroenterology, Queen Silvia Children’s Hospital, Gothenburg, Sweden

^j^Department of Gastroenterology, Sahlgrenska University Hospital, Gothenburg, Sweden

^k^Department of Health, Medicine and Caring Sciences, Linköping University and Department of Gastroenterology, Linköping, Sweden

^l^Department of Medicine, Höglandssjukhuset Eksjö, Region Jönköping County Council, Jönköping, Sweden

^m^Jönköping Academy for Improvement of Health and Welfare, Jönköping University, Jönköping, Sweden

^n^Department of Biomedical and Clinical Sciences, Linköping University, Linköping, Sweden and Clinical and Department of Surgery in Linköping, Region Östergötland, Linköping, Sweden

^o^Department of Molecular Medicine and Surgery, Karolinska Institutet, Stockholm, Sweden and Department of Pelvic Cancer, Karolinska University Hospital, Stockholm, Sweden

^p^Department of Clinical Science and Education, Södersjukhuset, Karolinska Institutet, Stockholm, Sweden and Division of Clinical Epidemiology, Department of Medicine Solna, Karolinska Institutet, Stockholm, Sweden and Sachs’ Children and Youth Hospital, Södersjukhuset, Stockholm, Sweden

All listed members in the SWIBREG study group are **Collaborators**, except from Pär Myrelid, Caroline Nordenvall and Ola Olén who also are **Authors**.
