# Supplementary material for: Declining rates of surgery in pediatric-onset inflammatory bowel disease: a Swedish nationwide register-based cohort study 2002-2022
Source: J Crohns Colitis. 2026 Sep 15;20(9):jjag127. doi: 10.1093/ecco-jcc/jjag127 (PMC13575933; doi:10.1093/ecco-jcc/jjag127)
Supplement: Supplementary file 1 [file jjag127_supplementary_data.docx]

**Appendix to Rolandsdotter et al.**

**DECLINING RATES OF SURGERY IN PEDIATRIC-ONSET INFLAMMATORY BOWEL DISEASE: A SWEDISH NATIONWIDE REGISTER-BASED COHORT STUDY 2002–2022**

Helena Rolandsdotter, Kári Kristjánsson, Åsa H Everhov, Caroline Nordenvall, *The SWIBREG study group**, Petter Malmborg, Pär Myrelid, Matilda Bräutigam, Jonas F Ludvigsson, Ola Olén

**Table of contents:**

***Title, authers***	***page1***

***Figure S1.*** *Overview of timeline for approval of biologic drugs (adult)s in Sweden*	***page 2***

***Table S1.*** *Overview of the data sources****.***

***Table S2a.*** *Codes defining Inflammatory Bowel Disease (IBD); Crohn’s disease,*	***page 3***

*ulcerative colitis and IBD-Unclassified and* ***Table S2b*** *codes defining comorbidity.*

***Table S3.*** *Nordic Medico-Statistics Committee (NOMESCO) Classification of Surgical*	***page 4*** *Procedures codes associated with inflammatory bowel disease*

***Table S4.*** *Anatomical Therapeutic Chemical (ATC) codes of inflammatory bowel disease therapies* ***page 5***

***Table S5.*** Descriptive characteristics at the end of follow-up in 8,200 incident cases of childhood-onset IBD in two cohorts (2002-2011 and 2012-2022)

***Figure S2*.** Age distribution at first PIBD diagnosis, first luminal IBD-surgery and the end of	***page 6***	follow-up (first surgery, death, emigration or December 31, 2022) in 8,200 incident cases in PIBD in the two time-cohorts. **Table S6**: Surgical procedures performed in 8,200 incident cases of childhood-onset (< 18 years) IBD in Sweden 2002-2022

***Figure S3 A-C.*** Cumulative incidence curves, with 95% confidence intervals, of (A) formation of ***page 7*** ileostoma; (B) formation of colostoma; (C) closure of stoma, in children with inflammatory bowel disease (2002-2011 and 2012-2022). ***Figure S4.*** *Cox regression analysis of perianal surgery in CD adjusted for sex and age at diagnosis*

***Table S7.*** Advanced therapy exposure before first luminal surgery among patients with first surgery ***page 8*** within 5 years of diagnosis, restricted to patients with at least 5 years of potential follow-up. ***Figure S5.*** *The cumulative incidence of colectomy in pediatric-onset UC and CD* *(subtype information based on all data available at end of follow-up) in two calendar periods; 2002-2011 and 2012-2022.*

***Figure S6.*** *Cumulative incidence curves for (A) segmental small bowel resection; (B)*	***page 9***	*segmental large bowel; (C) perianal surgery in pediatric-onset Crohn’s disease (2002-2011 and 2012-2022). Subtype definition (Crohn’s disease) based on all available information at end of follow-up.* **Figure S7.** The cumulative incidence in luminal childhood–onset surgery in two calendar periods; 2002-2011 and 2012-2019 with follow-up until 31 December 2019 (start of the Covid-19 pandemic)

***Figure S8*.** The cumulative incidence of colectomy in pediatric-onset UC and CD	***page 10***	in two calendar periods restricted to UC and CD patients with total colitis (E3-4) or Crohn’s colitis (L2) at start of follow-up. ***Table S8***. Cumulative Incidence at 1, 3, and 5 Years for colectomy in patients with pediatric-onset ulcerative colitis (UC) or Crohn’s disease (CD), restricted to total colitis (E3-4) or Crohn’s colitis (L2) at start of follow-up.

***References.*	*page 11***

**Figure S1.** Overview of timeline for approval of advanced therapies in Sweden for inflammatory bowel disease (Crohn’s disease (CD) or ulcerative colitis (UC) in adults.

**Table S1.** Overview of the data sources

| **Register name** | **Administrator** | **Type of data** | Start year |
| --- | --- | --- | --- |
| Total Population Register ^1^ | Statistics Sweden | Data on all persons registered in Sweden, including birth date, sex, place of residence (parish, municipality, or county, depending on availability of reference individuals), last immigration and emigration dates, and identity of parents/siblings | 1968- |
| Swedish National Patient Regist^2,3^ | Swedish National Board of Health and Welfare | Data from outpatient and inpatient visits in hospital-based care, including date of visit, primary and secondary diagnoses and procedures | 1964- hospitalizations  1997- outpatient surgery  2001- specialized outpatient visits |
| Swedish quality register for inflammatory bowel diseases SWIBREG ^4^ |  | Date of IBD diagnosis, Paris, complications, age at diagnosis, and start- and stop-date of biologic treatment | 2005- |
| Prescribed Drug Register ^5^ | Swedish National Board of Health and Welfare | Names and Anatomical Therapeutic Chemical (ATC) Classification of prescribed drugs dispensed from pharmacies. The coverage of the register is complete for prescriptions in ambulatory care (both specialist and primary care), while in-hospital drug treatment is generally not recorded on a patient level. | 2005- |

**Table S2a.** Codes defining Inflammatory Bowel Disease (IBD); Crohn’s disease, ulcerative colitis and IBD-Unclassified, and the Montreal/Paris classification of extent of ulcerative colitis and location of Crohn’s disease^6^ ^7^

|  | **Subtype of IBD** | **ICD-10** |
| --- | --- | --- |
|  |  | **1997-** |
| **Inflammatory bowel disease (IBD)**  **Ulcerative Colitis** | Ulcerative colitis (UC) | K51 |
|  | Crohn’s disease (CD) | K50 |
|  | Inflammatory bowel disease unclassified (IBD-U) | UC + CD, or K52.3 |
|  | **Extent according to Paris classification** |  |
|  | E1 = Ulcerative proctitis | K51.2 |
|  | E2 = Left-sided UC | K51.3; K51.5 |
|  | E3-E4 = Extensive UC | K51.0 |
|  | Extent not defined | K51.4; K51.8; K51.9 |
| **Crohn’s disease** | L1 = Small bowel disease; terminal ileitis (excluding colon engagement)  L2=Colon (excluding small bowel engagement)  L3= inflammation in both small and large bowel; Ileocecal Crohn’s disease | K50.0  K50.1  K50.8, K50.9 |

**Table S2b**. Codes defining comorbidity

| **Comorbidity** | **ICD_code** | **ATC_code** |
| --- | --- | --- |
| Ankylosing spondylitis | M45 |  |
| Cerebrovascular disease | I60-I69 |  |
| Chronic obstructive pulmonary disease | J41-J44 |  |
| Diabetes mellitus | E10-E14, O24 | A10 |
| Psoriasis arthritis | L405, M070, M071, M073 |  |
| Rheumatoid arthritis | M05, M060, M062, M063, M068, M069, M123 |  |
| Systemic lupus erythematosus | M320, M321, M328, M329 |  |
| Primary sclerosing cholangitis | K83.0 |  |
| Other extraintestinal manifestations (e.g., pyoderma gangrenosum, erythema  nodosum, Sweet syndrome, iridocyclitis, arthropathy in CD/UC, arthritis in CD/UC etc | L52, L88, L98.2, H20,  M07.4, M07.5, M07.6, M09.1, M09.2, M45, M460, M461, M468, M469, M139, M255 |  |

**ICD=International Classification of Diseases, version 10.
ATC=Anatomical Therapeutic Chemical (classification**

**Table S3.** Classification of Surgical Procedures codes

| **Procedure codes according to NOMESCO (The Nordic Medico-Statistical Committee)** | **7th revision*** |
| --- | --- |
| Colectomies |  |
| Colectomy and ileostomy with closure of the rectum | JFH10 |
| Laparoscopic colectomy and ileostomy | JFH11 |
| Other colectomy | JFH96 |
| Proctocolectomy with continent ileostomy | JFH40 |
| Proctocolectomy with ileostomy | JFH20 |
| Subtotal colectomy with end ileostomy | JFH00 |
| Laparoscopic colectomy with ileorectal anastomosis | JFH01 |
| Colectomy, rectal mucosectomy and ileal anastomosis without ileostomy | JFH30 |
| Colectomy, rectal mucosectomy and ileal anastomosis and ileostomy | JFH33 |
| Small bowel resection |  |
| Partial resection of small intestine | JFB00 |
| Laparoscopic partial resection of small intestine | JFB01 |
| Ileocaecal resection | JFB20 |
| Laparoscopic ileocecal resection | JFB21 |
| Large bowel resection |  |
| Right hemicolectomy | JFB30 |
| Laparoscopic right hemicolectomy | JFB31 |
| Resection of transverse colon  Laparoscopic resection of the colon transversum | JFB40  JFB41 |
| Left-sided hemicolectomy, laparoscopic | JFB43 JFB44 |
| Resection of the sigmoid colon, laparoscopicLaparoscopic resection of sigmoid colon | JFB46 JFB47 |
| Other colon resection, laparoscopic | JFB50 JFB51 |
| Resection of sigmoid colon with partial proctectomy | JFB53 |
| Laparoscopic resection of sigmoid colon with partial proctectomy | JFB54 |
| Resection of sigmoid colon with end colostomy with closure of distal stump | JFB60 |
| Lap. resection of sigm colon w. endcolostomy and closure of distal stump | JFB61 |
| Other resection of colon w proximal colostomy and closure of distal stump | JFB63 |
| Other intestinal resections |  |
| Other resection comprising small intestine and colon | JFB33 |
| Other laparoscopic resection comprising small intestine and colon | JFB34 |
| Other partial excision of intestine | JFB96 |
| Other laparoscopic partial excision of intestine | JFB97 |
| Strictureplasty to the colon | JFA63 |
| Closure of fistula of small intestine | JFA76 |
| Closure of colonic fistula | JFA86 |
| Other operation of intestine | JFW96 |
| Other laparoscopic operation of intestine | JFW97 |
| Formation of stoma | JFF |
| Partial excision of intestine | JFB |
| Excision of rectum | JGB |
| Operations on intestinal stoma or pouch | JFG |
| Strictureplasty in small intestine | JFA60 |
| Perianal surgery |  |
| Perianal incision and drainage | JHA00 |
| Excision of lesion of anal canal or perianal tissue | JHA20 |
| Dilatation of the anal sphincter | JHD00 |
| Lay open or excision of perianal fistula | JHD20 |
| Partial lay open or excision of perianal fistula (including seton placement) | JHD30 |
| Completion lay open or excision of perianal fistula | JHD33 |
| Excision of perianal fistula with advancement flap | JHD50 |
| Occlusion of perianal fistula with collagen plug | JHD60 |
| Occlusion of perianal fistula with fibrin glue | JHD63 |
| Stoma surgery |  |

Formation of ileostoma	JFF 10, JFF 11, JFF 13, JFF16, JFH 33, JFH10, JFH11

Formation of colostoma	JGB10, JGB11, JFF23, JFF24, JFF26, JFF27, JFF30, JFF31, JFB31, JFB60, JFB61, JFB63, JFB64

Formation of stoma of unknown location	JFF 96, JFF 97	Closure of stoma	JFG00, JFG10, JFG20, JFG23, JFG26, JFG29, JFG30, JFG33, JFG36, JFC40, JFC41

**Table S4.** Anatomical Therapeutic Chemical (ATC) codes study drugs

| **Drug category** | **Substance** | **ATC-code** |
| --- | --- | --- |
| **Conventional therapies** |  |  |
| Aminosalicylates | Mesalamine | A07EC02, A07EC03, A07EC04 |
|  | Sulfasalazine | A07EC01 |
| Immunomodulators | Azathioprine | L04AX0 1 |
|  | Mercaptopurine | L01BB02 |
|  | Methotrexate | L04AX03/L01BA01 |
|  |  |  |
| **Cortiocosteroids** |  |  |
| Corticosteroids, systemic | Betamethasone | H02AB01 |
|  | Methylprednisolone | H02AB04 |
|  | Prednisolone | H02AB06 |
|  | Prednisone | H02AB07 |
|  | Budesonide (oral formulation) | A07EA06 |
| Corticosteroids, local | Prednisolone, Hydrocortisone, Budesonide* | A07EA01, A07EA02, A07EA06 |
| **Advanced therapies** |  |  |
| **Biologics** |  |  |
| Anti-tumor necrosis factor-alfa | Infliximab | L04AB02 |
|  | Adalimumab | L04AB04 |
|  | Golimumab | L04AB06 |
| Anti- integrin | Vedolizumab | L04AG05 |
| Anti-IL12/23 | Ustekinumab | L04AC05 |
| **Small molecules** |  |  |
| Janus kinase inhibitors | Tofacitinib | L04AF01 |
|  | Upadacitinib | L04AF03 |
|  | Filgotinib | L04AF04 |
|  |  |  |

***** In the Prescribed drug register, the variable LFORM (drug formulation) needs to be some of the following: Rectal foam;
Rectal enema; Suppository.

**Table S5.** Descriptive characteristics at the end of follow-up in 8,200 cases of incident pediatric-onset inflammatory bowel disease (IBD) in 2002-2011 and 2012-2022.

| **Characteristics at end of follow-up** | **2002-2011** | **2012-2022** |
| --- | --- | --- |
| Total Patients (n, %) | 3,751 (100%) | 4,449 (100%) |
| Follow-up in years (median) | 13.9 | 4.3 |
| Ulcerative colitis (n, %) | 1,572 (42%) | 1,574 (35%) |
| Crohn’s disease (n, %) | 1,742 (46%) | 1,919 (43%) |
| IBD-Unclassified (n, %) | 437 (12%) | 956 (22%) |
| Exposed within 5yr to 5-ASA (n, %) | 3,219 (86%) | 3,391 (76%) |
| Exposed within 5yr to Immunomodulators (n, %) | 2,178 (58%) | 2,907 (65%) |
| Exposed within 5yr to Rectal Corticosteroids (n, %) | 1,821 (49%) | 2,061 (46%) |
| Exposed within 5yr to Systemic Corticosteroids (n, %) | 2,600 (69%) | 3,267 (73%) |
| Exposed within 5yr to Advanced therapies (n, %) | 419 (11%) | 1,051 (24%) |

**Figure S2**. Age distribution at first pediatric-onset IBD-diagnosis, age at first luminal surgical procedure, and age at the end of follow-up (first surgery, death, emigration or December 31, 2022) in 8,200 cases of incident pediatric-onset IBD, stratified by the two diagnostic periods.

**Table S6**: Surgical procedures performed in 8,200 incident cases of childhood-onset (< 18 years) inflammatory bowel disease (IBD) in Sweden 2002-2022

| **Characteristic** | **2002-2011** | **2012-2022** | **Overall** |
| --- | --- | --- | --- |
| **Total Patients (n, %)** | **3,751 (45.74%)** | **4,449 (54.26%)** | **8,200 (100%)** |
| Treated with any surgery | 953 (25.4%) | 523 (11.8%) | 1,476 (18,0%) |
| Treated with luminal surgery | 717 (19.1%) | 283 (6.6%) | 1,000 (12.2%) |
| **Surgery categories reported separately** |  |  |  |
| Colectomy | 319 (8.5%) | 117 (2.6%) | 436 (5.3%) |
| Segmental bowel resection small bowel | 332 (8.9%) | 135 (3.0%) | 467 (5.7%) |
| Segmental bowel resection large bowel | 89 (2.4%) | 24 (0.5%) | 113 (1.4%) |
| Perianal surgery | 434 (11.6%) | 286 (6.4%) | 720 (8.8%) |
| **Other bowel surgery, not reported separately** |  |  |  |
| Unspecified bowel resection (small and/or large bowel) | 40 (1.1%) | 8 (0.2%) | 48 (0.6%) |
| **Reconstructive surgery**  Ileorectal anastomosis (n, %) | 130 (3.5%) | 50 (1.1%) | 180 (2.2%) |
| Ileal pouch anal anastomosis (n, %) | 71 (1.9%) | 19 (0.4%) | 90 (1.1%) |
| Strictureplasty (n, %) | 18 (0.5%) | 0 (0%) | 18 (0.2%) |

**Figure S3 A-C.** Cumulative incidence curves, with 95% confidence intervals, of (A) formation of ileostoma; (B) formation of colostoma; (C) closure of stoma, in children with inflammatory bowel disease (2002-2011 and 2012-2022).

**Figure S4.** Hazard ratios for perianal surgery in Crohn’s disease (CD) after pediatric-onset CD diagnosis, estimated using a Cox proportional hazards model stratified by calendar period and adjusted for sex and age at diagnosis. Female and age group 15-17 years were used as reference categories.

**Table S7**

Advanced therapy exposure before first luminal surgery among patients with first luminal surgery within 5 years of diagnosis, restricted to patients with at least 5 years of potential follow-up.

| Characteristic | 2002-2011  N=364 | 2012-2022  N=143 |
| --- | --- | --- |
| Number of distinct advanced therapy agents prior to first surgery |  |  |
| 0 | 268 (74%) | 72 (50%) |
| 1 | 73 (20%) | 41 (29%) |
| ≥2 | 23 (6.3%) | 30 (21%) |

**Figure S5.** The cumulative incidence of colectomy in pediatric-onset UC and CD (**subtype information based on all data available at end of follow-up**) in two calendar periods; 2002-2011 and 2012-2022.

**Figure S6.** Cumulative incidence curves for (A) segmental small bowel resection; (B) segmental large bowel; (C) perianal surgery in pediatric-onset Crohn’s disease (**subtype information based on all available data at end of follow-up)** in two calendar periods 2002-2011 and 2012-2022.

**Figure S7.** The cumulative incidence in luminal childhood–onset surgery in two calendar periods; 2002-2011 and 2012-2019 with follow-up until 31 December 2019 (start of the **Covid-19 pandemic**).

**Figure S8**. The cumulative incidence of colectomy in pediatric-onset UC and CD in two calendar periods; 2002-2011 and 2012-2022, **restricted to UC and CD patients with total colitis (E3-4) or Crohn’s colitis (L2) at start of follow-up.**

**Table S8.** Cumulative Incidence at 1, 3, and 5 Years for colectomy in patients with pediatric-onset ulcerative colitis (UC) or Crohn’s disease (CD), restricted to total colitis (E3-4) or Crohn’s colitis (L2) at start of follow-up.

| Surgery Type | Diagnosis Period | 1 Year (%) | 3 Years (%) | 5 Years (%) |
| --- | --- | --- | --- | --- |
| Colectomy UC | 2002-2011 | 3.28 | 6.34 | 8.76 |
|  | 2012-2022 | 3.12 | 5.30 | 7.06 |
| Surgery Type | Diagnosis Period | 1 Year (%) | 3 Years (%) | 5 Years (%) |
| Colectomy CD | 2002-2011 | 0.70 | 1.05 | 2.09 |
|  | 2012-2022 | 0.41 | 0.93 | 1.59 |
|  |  |  |  |  |

**References**

1.	Ludvigsson JF, Almqvist C, Bonamy AK, et al. Registers of the Swedish total population and their use in medical research. Eur J Epidemiol 2016;31:125-36.

2.	Ludvigsson JF, Andersson E, Ekbom A, et al. External review and validation of the Swedish national inpatient register. BMC Public Health 2011;11:450.

3.	Everhov Å H, Frisell T, Osooli M, et al. Diagnostic accuracy in the Swedish national patient register: a review including diagnoses in the outpatient register. Eur J Epidemiol 2025.

4.	Ludvigsson JF, Andersson M, Bengtsson J, et al. Swedish Inflammatory Bowel Disease Register (SWIBREG) - a nationwide quality register. Scand J Gastroenterol 2019;54:1089-1101.

5.	Wettermark B, Hammar N, Fored CM, et al. The new Swedish Prescribed Drug Register--opportunities for pharmacoepidemiological research and experience from the first six months. Pharmacoepidemiol Drug Saf 2007;16:726-35.

6.	Satsangi J, Silverberg MS, Vermeire S, Colombel JF. The Montreal classification of inflammatory bowel disease: controversies, consensus, and implications. Gut 2006;55:749-53.

7.	Shrestha S, Olén O, Eriksson C, et al. The use of ICD codes to identify IBD subtypes and phenotypes of the Montreal classification in the Swedish National Patient Register. Scand J Gastroenterol 2020;55:430-435.
